# Assessment of the gene mosaicism burden in blood and its implications for immune disorders

**DOI:** 10.1038/s41598-021-92381-y

**Published:** 2021-06-21

**Authors:** Manuel Solís-Moruno, Anna Mensa-Vilaró, Laura Batlle-Masó, Irene Lobón, Núria Bonet, Tomàs Marquès-Bonet, Juan I. Aróstegui, Ferran Casals

**Affiliations:** 1 Institut de Biologia Evolutiva (CSIC-UPF), Departament de Ciències Experimentals i de la Salut, Universitat Pompeu Fabra, Doctor Aiguader 88, Barcelona, Spain; 2 Genomics Core Facility, Departament de Ciències Experimentals i de la Salut, Universitat Pompeu Fabra, Parc de Recerca Biomèdica de Barcelona, 08003 Barcelona, Spain; 3 Department of Immunology, Hospital Clínic, Barcelona, Spain; 4 Institut d’Investigacions Biomèdiques August Pi i Sunyer (IDIBAPS), Barcelona, Spain; 5 Catalan Institution of Research and Advanced Studies (ICREA), Passeig de Lluís Companys, 23, 08010 Barcelona, Spain; 6 CNAG-CRG, Centre for Genomic Regulation (CRG), Barcelona Institute of Science and Technology (BIST), Baldiri i Reixac 4, 08028 Barcelona, Spain; 7 Institut Català de Paleontologia Miquel Crusafont, Universitat Autònoma de Barcelona, Edifici ICTA-ICP, C/ Columnes S/N, Cerdanyola del Vallès, 08193 Barcelona, Spain; 8 Universitat de Barcelona, Barcelona, Spain; 9 Departament de Genètica, Microbiologia i Estadística, Facultat de Biologia, Universitat de Barcelona, Barcelona, Catalonia Spain

**Keywords:** Bioinformatics, Genomic analysis, Genetics, Immunology, Diseases, Medical research

## Abstract

There are increasing evidences showing the contribution of somatic genetic variants to non-cancer diseases. However, their detection using massive parallel sequencing methods still has important limitations. In addition, the relative importance and dynamics of somatic variation in healthy tissues are not fully understood. We performed high-depth whole-exome sequencing in 16 samples from patients with a previously determined pathogenic somatic variant for a primary immunodeficiency and tested different variant callers detection ability. Subsequently, we explored the load of somatic variants in the whole blood of these individuals and validated it by amplicon-based deep sequencing. Variant callers allowing low frequency read thresholds were able to detect most of the variants, even at very low frequencies in the tissue. The genetic load of somatic coding variants detectable in whole blood is low, ranging from 1 to 2 variants in our dataset, except for one case with 17 variants compatible with clonal haematopoiesis under genetic drift. Because of the ability we demonstrated to detect this type of genetic variation, and its relevant role in disorders such as primary immunodeficiencies, we suggest considering this model of gene mosaicism in future genetic studies and considering revisiting previous massive parallel sequencing data in patients with negative results.

## Introduction

The distribution and effect of somatic genetic variants in disease has been studied mostly in cancer. However, in the past years, they have also been identified in a wide spectrum of syndromes including neurological disorders as schizophrenia^1^, autism spectrum disorder^2^, Alzheimer^3–6^ or Huntington disease^7^, coronary heart disease and stroke^8^ and kidney diseases such as the Alport syndrome^9–11^. In fact, at least theoretically, all monogenic diseases could be originated by a postzygotic mutation and the resulting somatic mosaicism. In the field of immune-related diseases, a remarkable number of somatic variants have been described in monogenic autoinflammatory diseases^12–20^, and a recent work has shown its important contribution to these disorders and other primary immunodeficiencies (PIDs)^21^.

Understanding the relative abundance of somatic variants in health is critical to design efficient tools for mosaicism detection in disease studies. Different studies have measured the presence of somatic variation in normal tissues, most assessing the presence of mutations in cancer-driver genes, such as *NOTCH1* mutations, which undergo expansion through positive selection^22–24^. They reported the colonization of the tissue by mutant clones increasing with age and exposure to mutagenic agents (sun radiation, tobacco). Other studies, based on single cell^25^ or transcriptome analysis^26^ revealed tissue-specific patterns of somatic variant distribution, as well as negative selection of functional variants in non-cancer samples.

The detection of somatic variants from massive parallel sequencing (MPS) data presents some difficulties. Standard variant calling methods are based on the presence of germline heterozygous mutations in about 50% of the sequencing reads, and may fail to detect somatic variants in allelic imbalance and lower frequencies. Most of the algorithms developed for somatic variant analyses have been optimized for cancer studies where a tumour sample is compared with the healthy tissue from the same individual^27–30^. Of note, studies comparing the output of different variant callers have revealed low levels of overlap^29,30^. The tumour vs. healthy tissue approach is not suitable for somatic variant detection in mosaicisms, where the same postzygotic variant might be present in several tissues at similar frequencies. Alternatively, other variant calling tools can be applied to non-matched samples^31,32^. In this case, allelic imbalance thresholds will need to be relaxed to detect low frequency variants, at the cost of substantially increasing the number of candidate variants. Then, an adequate filtering strategy will be essential to differentiate sequencing artefacts from true genetic variants. These filters are based both on technical criteria to exclude sequencing or mapping errors and biological knowledge to restrict the analysis to a set of candidate regions. A validation step, such as amplicon-based deep sequencing (ADS), will be ultimately required to confirm the presence of a somatic variant and better determine its frequency.

In the present study we aim to assess the load of somatic coding variants in peripheral blood at detectable frequencies from MPS data, which is relevant to detect somatic causal variants in monogenic Mendelian diseases, in particular PIDs. These diseases represent a privileged scenario for the study of the somatic pathogenic variation because of the needed presence of the causal variant in blood, as well as probably in other easily accessible tissues, and the reported important contribution of somatic mutation in these disorders^21^. For this, we initially performed whole-exome sequencing (WES) in a total of 16 samples belonging to 12 individuals. All individuals carry a pathogenic and previously described somatic mutation related to a PID while one patient carries a germline variant. We then selected the best candidate somatic variants, based on read quality and mapping information, to be validated with ADS. With this analysis we have tested the ability to detect causal somatic variation in PID as well as estimated the actual number of functional coding variants in blood at detectable frequencies from WES data.

## Material and methods

### Ethical approval

Written informed consents for genetic analyses and participation in the study were obtained from each enrolled individual. The Ethics Committees of Hospital Clínic and Universitat Pompeu Fabra (reference number 7HCB/2019/0631), both located in Barcelona, approved the study, which was carried out in accordance with the principles and last amendments of the Declaration of Helsinki.

### Samples

The present study included both unique and matched samples from peripheral blood (PB), oral mucosa (OM) and urine (UR) for 12 individuals: (i) 11 unrelated PID patients carrying a pathogenic and previously described somatic variant, and ii) one of the descendants with the same pathogenic variant in germline status (Table 1). In eight individuals, the only analysed sample was PB (S2, S4a, S6, S8, S9, S10 and S11) or OM (S4). In four individuals, we analysed samples from paired tissues: from PB and OM in three patients (S1a-S1b, S3a-S3b and S5a-S5b) and, in the remaining patient, from PB and UR (S7a-S7b).

**Table 1:** Samples and mutations included in the study.

Sample	Coordinate (hg38)	Gene	Change in DNA	Change in protein	WES			ADS
					VAF (%)	DP/VD	Mean coverage	VAF (%)
S1a (PB)	chr1:247,424,492	*NLRP3*	c.1049C>T	p.Thr350Met	0	192/0	232	2.80
S1b (OM)	chr1:247,424,492	*NLRP3*	c.1049C>T	p.Thr350Met	7.22	97/7	153	6.90
S2 (PB)	chr1:247,424,357	*NLRP3*	c.914A>C	p.Asp305Ala	36.26	171/62	274	34.80
S3a (PB)	chr16:50,710,912	*NOD2*	c.1001G>A	p.Arg334Gln	10.13	592/60	220	9.40
S3b (OM)	chr16:50,710,912	*NOD2*	c.1001G>A	p.Arg334Gln	5.46	1171/64	349	4.90
S4a (PB)	chr16:50,710,912	*NOD2*	c.1001G>A	p.Arg334Gln	46.44	618/287	231	–
S4 (OM)	chr16:50,710,912	*NOD2*	c.1001G>A	p.Arg334Gln	5.21	576/30	179	8.50
S5a (PB)	chr1:247,425,355	*NLRP3*	c.1912C>G	p.Gln638Glu	19.67	422/83	318	18.40
S5b (OM)	chr1:247,425,355	*NLRP3*	c.1912C>G	p.Gln638Glu	8.72	390/34	274	6.00
S6 (PB)	chr1:247,424,367	*NLRP3*	c.924A>T	p.Gln308His	8.57	175/15	308	5.10
S7a (PB)	chrX:71,109,309	*IL2RG*	c.676C>T	p.Arg226Cys	18.75	192/36	247	17.80
S7b (UR)	chrX:71,109,309	*IL2RG*	c.676C>T	p.Arg226Cys	11.24	169/19	213	8.30
S8 (PB)	chr1:247,424,356	*NLRP3*	c.913G>A	p.Asp305Asn	8.00	125/10	234	7.20
S9 (PB)	chr16:50,710,912	*NOD2*	c.1001G>A	p.Arg334Gln	2.12	1038/22	312	2.70
S10 (PB)	chr14:35,007,365	*SRP54*	c.338G>T	p.Gly113Val	2.34	128/3	146	2.30
S11 (PB)	chr19:855,967	*ELANE*	c.607G>C	p.Gly203Arg	9.10	99/9	219	16.20

VAFs from ADS were extracted from a previous publication^21^.

*DP* total depth; *VD* variant depth.

All of the PID mutations are missense single nucleotide variants (SNVs), and are the disease causing mutation either in the proband or in its offspring, where they are germline variants. The range of variant allele frequencies (VAFs) for the somatic variants previously estimated by ADS^21^ ranges from 2.3 to 34.8%.

For patient S5 we included additional samples from urine, oral mucosa, whole blood (before and after anti-IL-1 treatment), and different cell type populations previously isolated by flow cytometry^20^: neutrophils, monocytes, B cells, T CD4 + cells and T CD8 + cells (all pre-treatment).

### Sequencing and genomic analysis

After DNA extraction, library preparation and exome capture were performed with the Nextera Rapid Capture kit (Illumina) according to the manufacturer’s instructions. The libraries were sequenced in a NextSeq Illumina platform in three High Output 2 × 150 paired-end cycles runs to a mean coverage of 245X. We used BWA-mem version 0.7.16a-r1181^33^ (https://github.com/lh3/bwa) to map the samples to the human reference genome hg38 (UCSC). We marked duplicated reads using Picard version 2.18.6 (https://github.com/broadinstitute/picard) MarkDuplicates and realigned indels using GATK’s version 3.7^35^ (https://github.com/broadgsa/gatk) IndelRealigner. We also performed base quality score recalibration using GATK’s BaseRecalibrator.

We used eight publicly available tools to call genetic variants: FreeBayes version 0.9.14-8-g1618f7e^34^ (https://github.com/freebayes/freebayes), HaplotypeCaller version 3.7^35^ (https://github.com/broadgsa/gatk), LoFreq version 2.1.2^36^ (https://github.com/CSB5/lofreq), MuTect2 version 3.7^35^ (https://github.com/broadgsa/gatk), SomVarIUS version 1.1^37^ (https://github.com/kylessmith/SomVarIUS), Strelka2 version 2.7.1^38^ (https://github.com/Illumina/strelka), VarDict version 1.0^39^ (https://github.com/AstraZeneca-NGS/VarDict) and VarScan2 version 2.4.3^40^ (https://sourceforge.net/projects/varscan/files/). FreeBayes and HaplotypeCaller are purely germline callers. SomVarIUS is a caller designed to detect somatic variants in unpaired samples. The rest of them support a single mode and a paired mode. Although in our study we were not analysing cancer samples, we tested the behaviour of variant callers’ paired mode in this context with the matched PB-OM and PB-UR samples. We used default parameters for all the callers except for VarScan2, where we lowered the allele frequency threshold of 20% and set the p-value to 1 to retrieve all the possible calls. For HaplotypeCaller, we first used the default ploidy parameter of 2 and next we considered other ploidy values: 4, 5, 6 and 10.

For variant calling, the manufacturer’s targeted regions were intersected with our VCF files to retrieve the on target genetic variants, and we restricted our analysis to these regions. We annotated the variants using SnpEff version 4.3t^41^ (https://sourceforge.net/projects/snpeff/files/) and SnpSift version 4.3t^42^ (https://sourceforge.net/projects/snpeff/files/). Using the database dbNSFP version 4.0b1a^43^, we added parameters of interest such as CADD score^44^, GERP score, ExAC^45^ and gnomAD allele frequencies. We also added two functional predictions, gene haploinsufficiency values^46^ and Residual Variation Intolerance Score (RVIS)^47^.

We performed ADS with rhAmpSeq from Integrated DNA Technologies (IDT, Coralville, USA) to validate the candidate somatic variants. We sequenced every selected position to a mean coverage > 20,000X in a NextSeq Illumina platform in a High Output 2 × 150 paired-end cycles run. The confirmed in blood plus 19 additional candidate somatic variants in S5 were analysed for validation in different tissues and cell population samples. They were sequenced in a MiSeq v3 run (2 × 300) to a final depth > 155,000X. We used BWA-mem version 0.7.16a-r1181 to map the fastq files to the human reference genome hg38 (UCSC). We then used pysam version 0.15.2 (https://github.com/pysam-developers/pysam) to count the number of reads supporting every allele, requiring a minimum mapping quality of 20 to calculate VAFs.

## Results

### Detection of somatic pathogenic variants from WES in PID patients

We performed WES in all DNA samples to a mean coverage of 245X (Table 1). The total number of genetic variants differs among the different callers (Supplementary Fig. S1), mostly because of VarDict and VarScan2, the two callers with relaxed allelic imbalance parameters, which called more than 200,000 variants each. These two callers also show high heterogeneity across samples, which correlates with sequencing depth, as expected in MPS experiments. The amount of overlapping variants across the different callers is uneven, especially for SomVarIUS, due to the low number of variants it calls. The number of concordant variants between VarDict and VarScan2 is also low, probably because VarDict calls 3–4 times the number of indels of Varscan2 and because of discrepancies calling low frequency variants (Supplementary Fig. S2).

**Figure 1:**
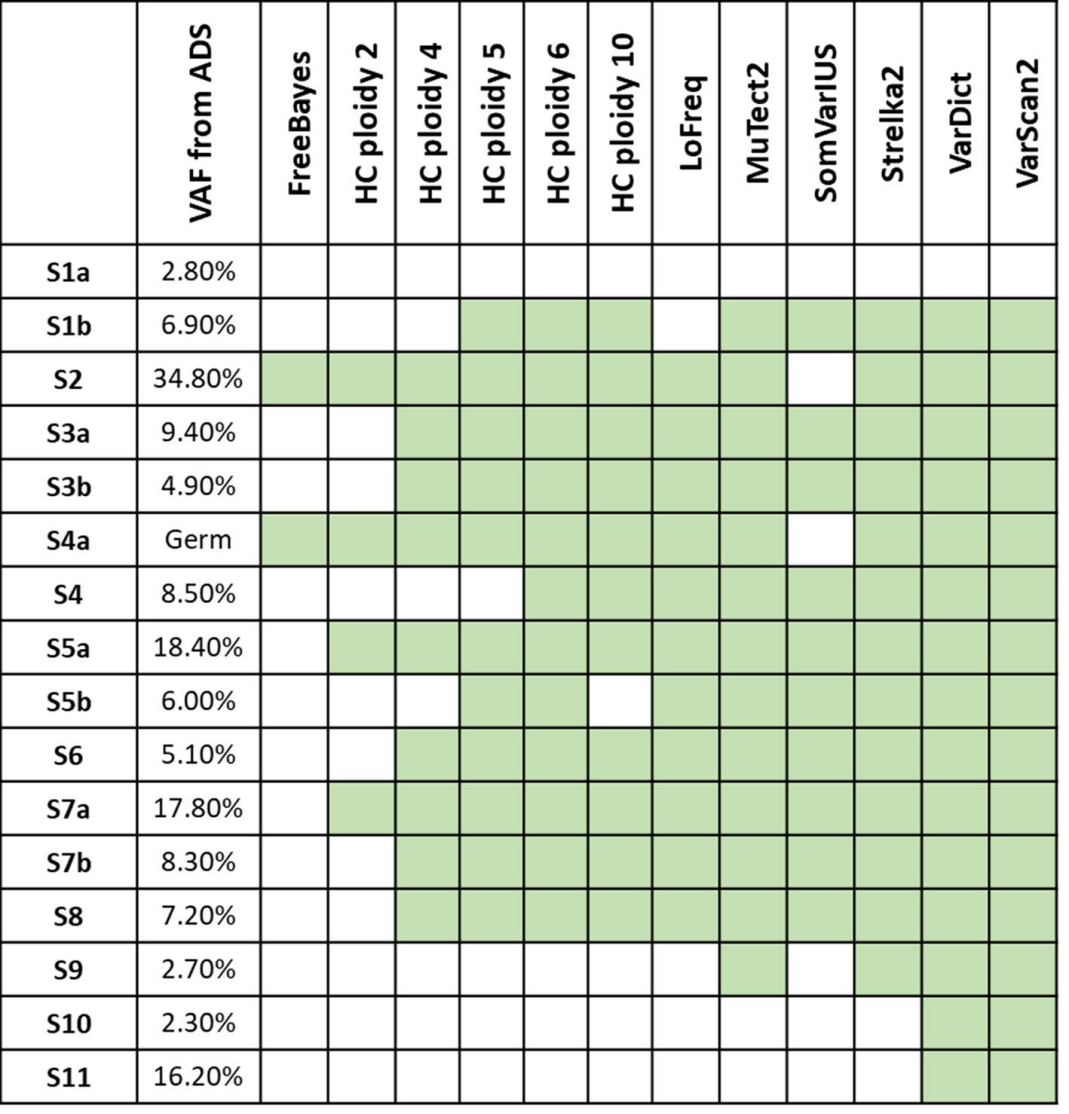
Previously reported causal somatic mutations detected by each variant caller (in green), assessed as the presence of the variant in the raw VCF files. The germline variant in S4a was detected in Strelka germline mode but not in the somatic one. All VAF were extracted from a previous publication^21^.

Figure 1 shows which known causal somatic variants (Table 1) are detected by each software. FreeBayes and HaplotypeCaller have the lowest detection ratios. For the rest, the ability of detection is similar and seems to depend on the frequency of the mutations, along with the coverage of the sample and the mapping quality. The S1a causal variant has not been called by any software, but visual inspection of the mapped reads revealed that none of them supported the alternative allele (Supplementary Fig. S3). Excluding it, VarDict and VarScan2 were able to detect all the causal variants. To increase the power of detection of HaplotypeCaller, we explored the effect of modifying the ploidy parameter. We used ploidy 2 (default), 4, 5, 6 and 10 in order to call variants with lower frequencies than expected in a germline scenario. This parameter is normally tuned when working with organisms with ploidies different than 2. For instance, decaploid plants have been reported^48,49^, and genotypes 0/0/0/0/0/0/0/0/0/1 are possible. This way, the increase of the ploidy parameter makes HaplotypeCaller more sensible to low frequency variants. The percentage of detected variants increased sequentially with the ploidy parameter, although some remained undetected. HaplotypeCaller seems to be sensitive to mapping quality as in the case of the *ELANE* region (Supplementary Fig. S4), where a variant with moderate frequency is not detected by this caller. Interestingly, we lost one variant using ploidy 10 while it was previously detected with ploidies 5 and 6 due to memory reasons (Fig. 1, expanded in Supplementary Fig. S5).

Next, we assessed the performance of the five variant callers including a paired mode in the four cases with available paired samples (S1, S3, S5 and S7), where the same variant is present in two tissues with different frequencies. As a general trend, there is no improvement of the detection rate when using the paired mode compared to the single mode, probably because of the small differences in allele frequency between tissues. The use of one or the other paired sample as cancer/healthy tissue does not seem to affect the capacity of detection. Again, VarDict and VarScan2 showed the best detection ratios (Supplementary Table S1).

### Filtering strategies for the identification of true causal variants

Once genetic variants have been called, a set of different filters is commonly applied to reduce the number of false positives. This is a crucial issue in the study of monogenic syndromes, where the aim is moving from the approximately 20,000 genetic variants identified in a typical WES to one or a few candidate variants. Relaxing or disabling the VAF filters to increase the ability to detect causal somatic variants, as we did in this study, produces an important increase of the number of mutations per individual, making this process highly recommended.

We evaluated the ability to identify the known pathogenic variants after applying the standard filters to the variants called by VarDict and VarScan2, the most successful programs in calling them (Fig. 1). We started by intersecting the two VCF files for every individual, given that in all cases the true variants were retained by both of them. Next, we applied a set of additional filters sequentially (see below), checking in every step if the causal variant was retained or filtered out (Table 2). First, we filtered out SNPs located 6 bp around indels. Second, as suggested previously^50^, we restricted our analysis to the 1000 Genomes Project strict mask filter. Third, we required the positions to be covered by, at least, 50 reads (DP > 50) and to show a minimum quality value of 25 (QUAL > 25). Fourth, we only kept loss of function and missense variants. Fifth, we applied a stringent population allele frequency threshold of 0.001 in gnomAD. With a high probability, a somatic variant will be absent in the population because of its de novo nature, although the possibility of having a recurrent mutation cannot be excluded. Sixth, following the recommendations in the literature, we kept variants with a likely damaging predicted effect (CADD > 15^44^) and a high evolutionary conservation score, as an indicator of its functional importance (GERP > 2^51^). Seventh, we required at least three reads supporting the alternative allele (VD ≥ 3) in every call. Finally, we used the list of 333 genes of the International Union Of Immunological Societies (IUIS, updated in February 2018)^52^ as a set of candidate genes for PIDs. Excluding the causal somatic variant of sample S1a, which was not detected in the sequencing process, 13 out of the 14 somatic mutations were included in the final list of candidate variants. The remaining one (S6), was filtered out because of a GERP value lower than 2.

**Table 2:** Numer of called variants and after sequential variant filtering process for each sample.

Filtering	S1a	S1b	S2	S3a	S3b	S4	S5a	S5b	S6	S7a	S7b	S8	S9	S10	S11
On target (VarDict − VarScan2)	298,250 173,072	363,209 101,637	239,526 266,542	286,381 200,507	241,135 453,494	382,519 119,025	231,469 263,604	312,808 245,467	276,040 273,296	293,494 176,720	315,825 171,718	274,664 317,568	191,603 720,791	223,731 261,383	302,540 172,629
Intersection	48,715	44,246	49,871	51,066	70,617	49,399	52,849	61,889	62,644	53,951	51,705	58,201	64,366	50,684	53,268
6pb indels	48,187	43,598	49,246	50,477	69,885	48,451	52,286	61,126	61,954	53,382	50,880	57,646	63,757	50,157	52,621
1000G mask	35,864	31,825	37,231	37,437	55,243	35,592	39,619	47,635	48,200	40,621	38,732	44,829	50,966	37,963	39,983
DP > 50	34,091	27,692	36,459	35,706	54,052	31,989	38,734	45,779	46,668	37,906	35,927	42,349	49,958	33,123	36,816
QUAL > 25	33,771	27,346	35,991	35,272	53,184	31,542	38,295	45,035	45,851	37,388	35,353	41,439	48,835	32,584	36,282
LoF & missense	18,476	15,154	19,998	18,929	31,534	18,100	21,148	26,962	27,596	22,103	20,585	24,283	28,888	18,518	21,472
gnomAD < 0.001	12,135	10,119	13,562	11,980	24,001	12,871	14,427	20,553	21,178	16,281	14,781	17,977	21,910	12,711	15,634
CADD > 15	9,035	7,904	10,085	8,864	19,044	9,994	10,828	16,271	**16,808**	12,662	11,077	13,887	17,086	9,547	12,155
GERP > 2	7,787	6,771	8,703	7,604	16,486	8,582	9,374	13,979	14,498	10,953	9,528	11,976	14,633	8,161	10,409
VD ≥ 3	6,977	6,086	7,446	6,528	14,473	7,720	8,560	12,509	13,231	9,764	8,181	9,719	11,024	5,162	8,991
Candidate genes	174	**177**	**174**	**172**	**319**	**219**	**187**	**276**	275	**226**	**255**	**263**	**243**	**144**	**229**

The last step where the causal somatic variant is retained is shown in bold.

### Mosaicism abundance detection in whole blood

**Figure 2:**
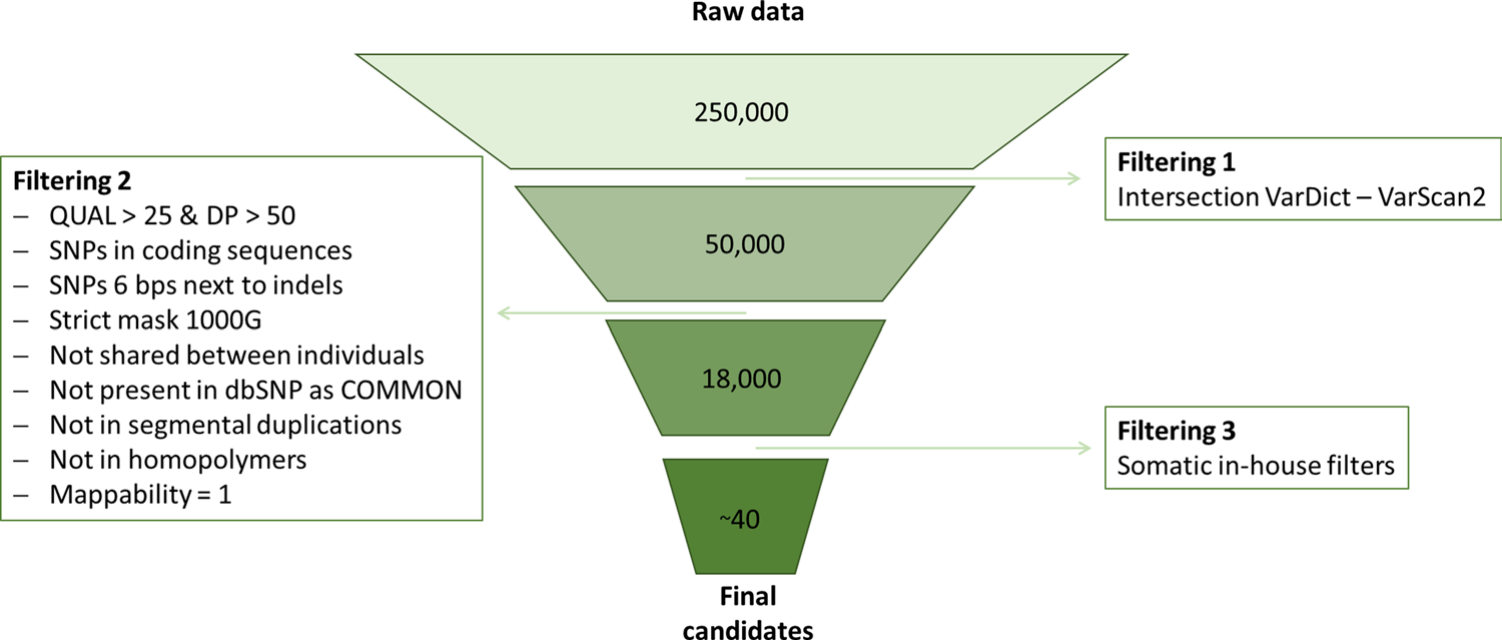
Filtering process followed to obtain somatic candidate variants. We got around 40 variants per blood sample that we then experimentally validated by ADS.

As mentioned above, the consideration of genetic variants deviating from the approximate expectation of 50% read frequency increases substantially the number of called variants. In the previous analyses we assessed how many of the true causal variants in 11 PID samples were detected. Now we wonder what proportion of the called variants in these samples corresponds to real postzygotic mutations, and not to sequencing, mapping or calling errors. We restricted the analysis to coding variants, more prone to have a functional impact and to be related to monogenic disorders. For this, we applied the following filters to select the variants more plausible to be validated as true: we intersected the SNPs called by VarDict and VarScan2, removed SNPs located 6 bps around indels, applied 1000G strict mask, required a minimum depth of 50 and a minimum quality of 25, removed variants classified as common in dbSNP and those shared among samples in the study, removed SNPs located within homopolymers, and removed SNPs in positions where the mappability was not perfect. We also performed a binomial test to exclude potential heterozygous mutations, to estimate the possibility of the observed number of reads supporting the alternative allele given the total number of reads. We finally required a minimum number of reads supporting the alternative allele of 7, due to the large number of variants below this threshold in our dataset (Supplementary Fig. S6). After this filtering, we moved from the approximately 250,000 variants called per individual to around 40. (Fig. 2), representing a total of 461 candidate somatic variants (Supplementary Table S2) for the 11 blood samples. 327 (70%) of the variants were missense, while 92 (20%) were synonymous and 19 (4%) were stop-gain. The remaining 23 variants were annotated as structural interaction variants and splice variants. Remarkably 30 of the variants were located in zinc finger proteins, 20 of them located in chromosome 19, and none of them were validated.

The 461 candidate variants were analysed by ADS with the rhAmpSeq technology (see Methods). All candidate positions were resequenced in the individual in which they were called and in the rest of individuals, plus two healthy individuals as controls. The average coverage per position was 22,500X (max = 272,401, min = 0, sd = 21,296). The overall validation ratio was very low. For five individuals (S6, S7, S8, S9 and S10), only the initial pathogenic variant was validated, with none of the other additional candidate variants confirmed. In other six individuals, including the individual with no somatic variants (S4a), we validated one additional variant: one missense variant in *ODF2* (S1), *SHISHA2* (S2), *STRIP1* (S3) and *IL2RG* (S11), and one synonymous variant in *CACNAS1* (S4) and *ROBO4* (S11). Of note, in patient S5 we validated a total of eleven variants: seven missense, being one of them the causal variant in *NLRP3*, and four synonymous. The twelve variants seemed to cluster in two frequency groups: one with variants of about 25% (including the pathogenic variant) and other with variants about 4.5% (Supplementary Table S2).

### Cell type distribution of somatic variants in S5 patient

Given the high number of validated somatic variants in patient S5, we expanded the analysis selecting nine additional candidate genetic variants. These variants were analysed for validation, along with the twelve previously confirmed, both in the whole blood sample and different cell populations separated by flow cytometry^20^ (Table 3). We also added a whole blood DNA extraction obtained after the anti-IL-1 treatment this patient received. In this experiment, the average coverage per position was 158,000X (max = 484,219, min = 16,689, sd = 80,940). We considered that a somatic variant was validated in a given cell type or tissue when the proportion of reads supporting the alternative allele was above 0.30%, a value close to the average error type of sequencing by synthesis technologies, which also varies with features such as sequence context or the specific nucleotide change^53,54^. Six of the nine new genetic variants were validated, with one (chr7:157,614,060) being a germline variant according to its frequency (Table 3).

**Table 3:** VAF of the 20 somatic candidate variants studied in S5 patient.

Chr	Position	Gene	Type	Whole blood	Whole blood post	Urine	Oral mucosa	Neutrophils	Monocytes	B cells	TCD4	TCD8	Control1	Control2	Validated
chr1	153,003,501	*SPRR3*	Missense	7.7216	3.7419	7.3138	1.6876	7.165	9.5398	**0.1088**	**0.1049**	**0.0976**	**0.0832**	**0.1081**	YES
chr1	247,425,355	*NLRP3*	Missense	24.6228	12.4922	25.3693	7.0918	24.3422	33.993	**0.0825**	**0.0794**	**0.0787**	**0.0437**	**0.0571**	YES
chr2	24,300,108	*ITSN2*	Missense	4.7359	3.4122	5.0154	1.8296	5.6246	2.2179	**0.3675**	**0.1288**	**0.1415**	**0.1035**	**0.1628**	YES
chr2	209,888,127	*UNC80*	Synonimous	26.5932	13.452	28.2002	6.9989	26.8757	34.7143	**0.2961**	**0.2798**	**0.2896**	**0.2373**	**0.2993**	YES
chr2	219,251,622	*TUBA4A*	Synonimous	4.5508	3.2531	4.6625	1.3055	5.3645	1.9301	**0.2923**	**0.1629**	**0.1553**	**0.0815**	**0.1322**	YES
chr3	52,913,506	*SFMBT1*	Missense	1.4477	1.2013	1.3699	0.4748	1.7193	1.453	2.5658	**0.1158**	**0.1569**	**0.062**	**0.068**	YES
chr4	143,695,587	*FREM3*	Missense	24.3856	12.176	24.6201	6.1085	23.192	31.5614	**0.167**	**0.1334**	**0.1238**	**0.0887**	**0.1295**	YES
chr4	165,059,454	*TRIM60-TMEM192*	Intergenic	**0.073**	**0.0596**	**0.0601**	**0.0673**	**0.0659**	**0.0515**	**0.0646**	**0.0892**	**0.0532**	**0.0676**	**0.0533**	**NO**
chr6	36,270,463	*PNPLA1*	Missense	4.8759	4.0553	5.5917	1.7713	6.4759	2.6609	**0.3096**	**0.0835**	**0.0768**	**0.0657**	**0.0772**	YES
chr6	52,082,518	*PKHD1*	Missense	26.3027	12.7771	27.3696	6.8415	26.0161	35.482	**0.1139**	**0.1225**	**0.0916**	**0.0923**	**0.111**	YES
chr6	151,349,029	*AKAP12*	Missense	1.496	1.2648	1.6739	0.4203	1.8367	1.2469	2.7175	**0.0769**	**0.1867**	**0.0872**	**0.0911**	YES
chr7	157,614,060	*PTPRN2*	Intronic	47.7712	48.8889	46.9676	46.2124	49.503	48.0925	48.1535	43.1712	46.6121	**0.0656**	**0.0674**	NO
chr9	91,410,553	*NFIL3*	Missense	**0.1427**	**0.1298**	**0.127**	**0.1367**	**0.109**	**0.0848**	**0.1351**	**0.118**	**0.1174**	**0.129**	**0.1463**	**NO**
chr11	111,853,480	*ALG9*	Synonimous	4.2723	3.1181	4.6411	1.3842	5.2542	2.2404	**0.2136**	**0.2158**	**0.229**	**0.1434**	**0.2365**	YES
chr12	128,705,237	*TMEM132C*	Missense	2.4998	1.2072	2.4715	0.5706	2.2107	3.4175	**0.1087**	**0.0703**	**0.0635**	**0.081**	**0.0814**	YES
chr13	24,912,928	*CENPJ*	Missense	1.5069	1.1153	1.8446	0.6136	1.8944	0.9268	1.2501	**0.1418**	**0.2347**	**0.0655**	**0.0754**	YES
chr17	50,840,691	*WFIKKN2*	Missense	4.1908	2.7357	4.4201	1.3373	4.4152	1.9862	**0.1779**	**0.1208**	**0.1042**	**0.0674**	**0.0797**	YES
chr19	16,529,871	*CHERP*	Synonimous	4.6041	2.2909	4.4651	1.1656	4.6677	5.4904	**0.1021**	**0.1105**	**0.0973**	**0.0697**	**0.0885**	YES
chr20	13,915,139	*SEL1L2*	Intronic	24.5724	11.9959	24.142	6.1982	23.3935	33.2716	**0.0564**	**0.0719**	**0.0733**	**0.0562**	**0.0869**	YES
chrX	71,537,899	*OGT*	Missense	49.978	25.1551	48.1124	12.4303	48.9245	32.2739	**0.2688**	**0.275**	**0.2301**	**0.1665**	**0.2497**	YES

In bold, values below the sequencing error threshold.

**Figure 3:**
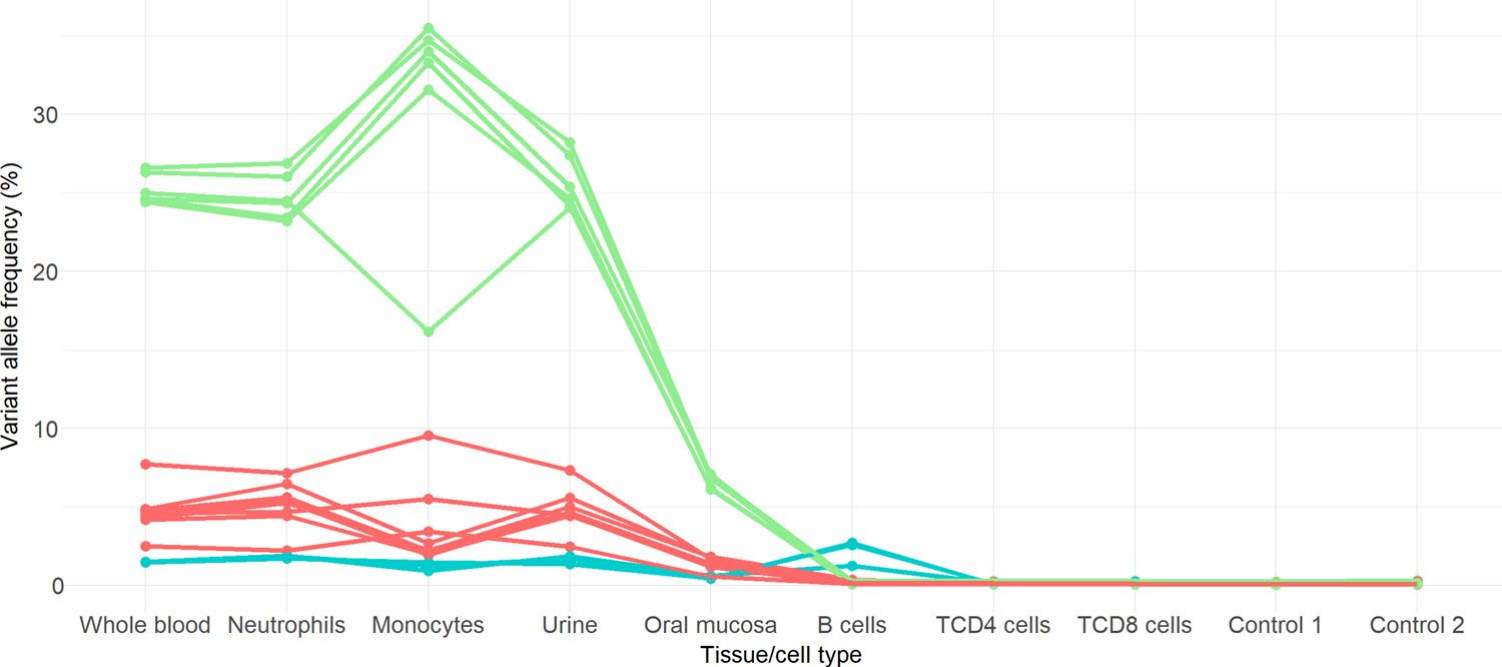
VAF of validated somatic variants in S5 patient per tissue and cell type. Green is used for the group of variants with higher VAF (around 24%), red for those with intermediate VAF (around 4%) and blue for those with low VAF (around 1.5%, the only group present in B cells). Of note, there is one variant in the X chromosome whose frequency has been divided by 2 in order to visualize it grouped with the others.

Overall, we detected 17 somatic variants in this patient, 16 protein coding and one intronic (Table 3), now clustered in three groups with similar VAFs around 24%, 4.5% and 1.5% in whole-blood pre-treatment (Fig. 3) and cell type distribution. VAFs changes across different cell types and tissues are coordinated within each group, being the two main groups only present in the myeloid line as well as in urine and cell mucosa, but absent in the lymphoid line. In general, we found higher allele frequencies in monocytes and lower in oral mucosa. The presence of the somatic variants in oral mucosa and urine was produced by leukocyte infiltration, which was detected by flow cytometry^20^. On the other hand, the lowest VAF group of variants are detected in myeloid cells and B cells, but not in T cells. The VAF of all the somatic variants is reduced in the whole-blood sample after the anti-IL-1 treatment (Whole blood 3 post, in Table 3). This decrease is more important for the variants restricted to the myeloid line, and it is likely observed because of the increased proliferation of inflammatory cells, which is now controlled with the treatment^20^.

## Discussion

We performed WES of DNA samples from patients with PIDs, carrying variable degree of gene mosaicism and assessed the ability to detect the somatic causal genetic variants by using different tools. Among the eight variant callers tested, VarDict and VarScan showed the higher detection rates of the causal somatic variants. The rest of the callers designed for somatic variant detection (MuTect2, SomVarIUS and Strelka2) mainly showed some limitations with the lower frequency variants at lower coverage. FreeBayes and HaplotypeCaller, designed for germline variant detection, failed to detect most of the somatic mutations. However, the performance of HaplotypeCaller increases when modifying the ploidy parameter, devised for non-diploid organisms and which allowed retrieving variants with less frequency than the expected 50% in the germline. Of interest, the efficiency of the five callers including a paired mode did not increase when using paired samples, probably because of the small frequency difference between the two samples carrying the same mutation.

Allele frequency is the main limitation for calling a somatic variant, with the risk of non-capturing the mutation because of its low frequency and/or insufficient coverage. To capture these low frequency variants, sequencing depths should ideally be higher than the commonly average depths achieved in WES studies (60-100X). However, the average coverage value might not be informative enough on the sequencing performance for all genomic regions, given the non-uniformity of the capture process. The use of new metrics including this information has been proposed^55^, which should help to reduce false-negative results. As an example, the *NOD2* region is clearly captured more efficiently than the *NLRP3* region in our study (Table 1). On the other side, only a few reads supporting the alternative allele seems enough to detect the variant, with as few as 3 (out of 128) for the S10 variant or 7 (out of 97) for the S1b variant (Table 1). Thus, an increase of the sequencing depth to 100-200X is recommendable in cases in which somatic variation is suspected. Higher coverage facilitates the detection of very low frequency variants, but increases the risk of enlarging the list of candidate variants because of approaching the error rate of MPS technologies^56^.

Genetic studies usually implement a set of filters to reduce the number of candidate variants to the causal one or to a small group. This process is a trade-off between reducing the number of false positives (either sequencing or mapping artefacts, and non-causal variants) and false negatives (called but filtered true causal variants). At the risk of missing the causal variant, these filters are essential to determine, at least, a reduced list of candidate genes for monogenic syndromes. In the case of studies like this, where the relaxation of allele frequency thresholds generates a list of up to hundreds of thousands of variants per sample (Supplementary Fig. S1), this step can be especially critical. After applying commonly used filtering parameters both for sequencing and biological features, only the causal variant in one patient was discarded because of low conservation score (GERP for S6 causal variant: -8.07). In the case of applying more stringent filters, two more variants (S1b and S5a-b) would be missed due to GERP score vale lower than 4^57,58^. On the other side, only S6 causal variant would not pass a CADD threshold of 20.

The final number of candidate genetic variants exceeds by about ten times the number of variants in studies analysing germline variants. Considering the IUIS list of 333 candidate genes for PIDs, this is still quite high, with approximately 0.5 variants per gene in each individual. Therefore, it seems recommendable to restrict the analysis to a reduced set of candidate genes according to the clinical phenotype of each patient. Alternatively, the use of some gene features could also help to reduce the list of candidate variants if there is not any a priori clear candidate. Several gene indexes have been developed to measure their possible contribution to human disease. Among them, haploinsufficiency predictions could seem useful for identifying candidate genes in a somatic variant disease model expecting to follow a dominant inheritance pattern. However, all the genes with somatic causal variants included in this study show haploinsufficiency values below the consensus threshold of 0.5, with *NLRP3*, a gene that is proven to be mutated in different autoinflammatory diseases^59^, showing the highest value of 0.465. In contrast, *NLRP3* has been reported as a gene with a high level of intolerance to functional variation (RVIS = − 0.95, in the top 9.38% of genes)^21^.

It is important to consider that exome sequencing was performed in DNA samples obtained from peripheral blood. Therefore, only somatic variants present in the major cell populations in blood can be detected. Neutrophils represent more than half of the nucleated blood cells (55–75%) in healthy individuals, while lymphocytes represent around 20% (from which T cells are ~ 70%, B cells are just ~ 20%, and NK cells ~ 10%)^60^. Thus, for early postzygotic mutations, the capacity of detection will most probably not be affected by the cell type implicated in the disorder, since the variant will have similar frequencies in all cell populations. In contrast, for later onset mutations restricted to particular lineages, the mutation will only be detectable if present in the major cell populations of the analysed tissue. Therefore, for immune disorders, the probability of detecting a causal variant from whole-blood extraction analysis will be much higher in those produced by alteration in the myeloid cells, such as in autoinflammatory disorders, than in the lymphoid cells. This fact can partially explain the larger number of reported cases in autoinflammatory disorders^21^ compared to other PIDs, as well as the lack of success in the identification of somatic variants in lymphoid immunodeficiencies such as CVID^61^. In these latter situations, it is expected that a big proportion of somatic causal variants would only be detectable if the analysis is restricted to particular cell types. Thus, cell subsets isolation can be essential to the identification and/or the validation of somatic genetic variants in these less represented cell types.

Beyond the detection of the known causal variants, the detected load of coding variants per exome was very low. Except for S5, all the individuals carry none or only one somatic variant additionally to the causal variant. The vast majority of candidate variants were false positives, even if they passed the mapping and quality filters. Comparing our results to other studies is not straightforward because of the differences in the methodologies used and the scanned VAFs, as well as the conceptual approach and targeted regions (see “Introduction”). A whole-genome sequencing (WGS) data analysis of 11,262 blood samples revealed a median number of three mosaic mutations for younger individuals, increasing after 35–45 years of age, and considering 20 somatic variants as the threshold for clonal expansion, that affecting 12.5% of the individuals^62^. Although the minimum detectable VAF of the study was limited because of the 34.8X mean coverage, the results seem concordant with the low number of somatic variants described in our WES deep sequencing approach. In addition to scanning a wide range of VAFs, we validated our results by ADS, which confirmed the low number of somatic coding variants detectable in blood. At a finer level, the total number of somatic variants per cell has been estimated in single-cell studies^25,26^, although most of this variation would remain undetected when the whole tissue is analysed. In fact, when much lower frequencies have been scanned (VAF ≥ 0.0001), it has been shown that clonal haematopoiesis is present in up to 97% of middle-aged people^63^. However, in absence of positive selection on a given mutation, only those that occurred earlier would reach detectable frequencies.

We identified a particular patient with an excess of validated variants compared to the others. S5 is the oldest individual of our dataset (64 years old), although another individual of similar age was also included in this study. Especially for the higher VAF group of five variants (which includes the causal one in *NLRP3*), the frequency pattern is quite uniform, except for one of the variants in chromosome X (chrX:71,537,899), with lower frequency in monocytes. The presence of the genetic variants in the lowest frequency cluster in cells of the myeloid lineage and in B cells, but not in T cells, could be explained by its origin in adult hematopoietic stem cells generating multineage outputs^64^. Because of the seemingly aggrupation in three different clusters of frequencies and cell type distribution, we propose simultaneous occurrence and clonal expansion as the most parsimonious explanation. However, none of the genes with somatic variants in S5 (Table 3) seems to be related with cellular proliferation that could be linked to an adaptive advantage of a clone of cells, and we also discarded the presence of additional candidate variant in *DNMT3A*, *TET2* and *ASXL1* genes, known to be implicated in hematologic malignancies^8,65^. In fact, in the aforementioned study of WGS of 11,262 individuals^62^ only 12.6% of the cases of clonal haematopoiesis had detectable cancer driver mutations. Thus, on the rest of cases as well as for S5, clonal haematopoiesis could be produced by genetic drift, as suggested in simulation analysis^66^. In contrast, a recent study^67^ proposes positive selection being the major driving force of clonal haematopoiesis, and that it would take more than 2000 years for a mutation to reach a VAF > 1% by only drift. However, our results do not seem to fit to this explanation, because of the abovementioned gene location as well as the presence of synonymous and intronic variants.

Finally, although we believe that our study contributes to the understanding of the burden of functional somatic mutations in blood and provides some practical advice on its detection, we would like to acknowledge some limitations of our approach. Allele frequency and sequencing depth are the two main limiting factors to detect a somatic variant as shown in our case by the failure to detect a variant with VAF < 3%. Also, the number of genetic variants depends on the selected software, that show a limited level of overlapping among them. In this sense, we recommend an inclusive strategy by using the less stringent callers or parameters, followed by a filtering strategy based on sequencing and mapping features. However, even by using stringent filters, the capacity of detection of causal variants will be mostly limited to previously known candidate or related genes, given the excessive number of variants when considering the whole exome. Gene functional relevance or mutation tolerance indexes could be used to reduce the number of candidate genes, but they also show limited applicability. Of importance, we also acknowledge the limitations derived from the small size of our cohort which, while allowing the study of somatic variant discovery, makes it difficult to draw conclusions in terms of dynamics of somatic variation.

## Conclusions

The detectable genetic load of somatic coding variants in blood is low. A moderate increase of the commonly achieved depths in exome sequencing analyses can be enough to detect most of these variants at frequencies above the technology error rate, for which we recommend using variant callers sensitive to low VAF. Of importance, the high proportion of false positives makes mandatory their validation which will also provide a better estimation of the VAF. Given both the feasibility of this approach and the reported contribution of gene mosaicism to PIDs^21^, we think that this model should be considered in future sequencing studies. It can be of special interest for those disorders related to major cell populations in blood, such as autoinflammatory diseases. We also suggest reanalysing data of undiagnosed patients, especially those where the inheritance pattern in the pedigree and/or the clinical features of the patient might fit this model. Because of the high number of possible somatic variants called per individual, even after applying stringent filters, it is advisable to restrict the analysis to a set of candidate genes defined according to the clinical phenotype. Finally, our results are in agreement with the existence of clonal haematopoiesis produced by drift, and that can be related to non-cancer disorders.

## Supplementary Information

Supplementary Information 1.

Supplementary Information 2.

## Data Availability

The datasets generated during and analysed during the current study are available in the European Nucleotide Archive (ENA) repository under accession code PRJEB44742 (https://www.ebi.ac.uk/ena/browser/view/PRJEB44742).
