# Publisher Correction: Assessment of the gene mosaicism burden in blood and its implications for immune disorders

**DOI:** 10.1038/s41598-021-96172-3

**Published:** 2021-08-17

**Authors:** Manuel Solís-Moruno, Anna Mensa-Vilaró, Laura Batlle-Masó, Irene Lobón, Núria Bonet, Tomàs Marquès-Bonet, Juan I. Aróstegui, Ferran Casals

**Affiliations:** 1 Institut de Biologia Evolutiva (CSIC UPF), Departament de Ciències Experimentals i de la Salut, Universitat Pompeu Fabra, Doctor Aiguader 88, Barcelona, Spain; 2 Genomics Core Facility, Departament de Ciències Experimentals i de la Salut, Universitat Pompeu Fabra, Parc de Recerca Biomèdica de Barcelona, 08003 Barcelona, Spain; 3 Department of Immunology, Hospital Clínic, Barcelona, Spain; 4 Institut d’Investigacions Biomèdiques August Pi i Sunyer (IDIBAPS), Barcelona, Spain; 5 Catalan Institution of Research and Advanced Studies (ICREA), Passeig de Lluís Companys, 23, 08010 Barcelona, Spain; 6 CNAG CRG, Centre for Genomic Regulation (CRG), Barcelona Institute of Science and Technology (BIST), Baldiri i Reixac 4, 08028 Barcelona, Spain; 7 Institut Català de Paleontologia Miquel Crusafont, Universitat Autònoma de Barcelona, Edifci ICTA ICP, C/Columnes S/N, Cerdanyola del Vallès, 08193 Barcelona, Spain; 8 Universitat de Barcelona, Barcelona, Spain; 9 Departament de Genètica, Microbiologia i Estadística, Facultat de Biologia, Universitat de Barcelona, Barcelona, Catalonia Spain

Correction to: *Scientific Reports* https://doi.org/10.1038/s41598-021-92381-y, published online 21 June 2021

The original PDF version of this Article omitted an affiliation for Ferran Casals. The correct affiliations for Ferran Casals are listed below:

Genomics Core Facility, Departament de Ciències Experimentals i de la Salut, Universitat Pompeu Fabra, Parc de Recerca Biomèdica de Barcelona, 08003, Barcelona, Spain

Departament de Genètica, Microbiologia i Estadística, Facultat de Biologia, Universitat de Barcelona, Barcelona, Catalonia, Spain

This error has now been corrected in the PDF version of the Article; the HTML version was correct from the time of publication.

