# Supplementary material for: Assessment of the gene mosaicism burden in blood and its implications for immune disorders
Source: Sci Rep. 2021 Jun 21;11:12940. doi: 10.1038/s41598-021-92381-y (PMC8217568; doi:10.1038/s41598-021-92381-y)
Supplement: Supplementary file 1 — Supplementary Information 1. [file 41598_2021_92381_MOESM1_ESM.docx]

**Assessment of the gene mosaicism burden in blood and its implications for immune disorders**

Manuel Solís-Moruno^1,2^, Anna Mensa-Vilaró^3,4^, Laura Batlle-Masó^1,2^, Irene Lobón^1^, Núria Bonet^2^, Tomàs Marquès-Bonet^1,5,6,7^, Juan I. Aróstegui^3,4,8^, Ferran Casals^2^

^1^ Institut de Biologia Evolutiva (CSIC-UPF), Departament de Ciències Experimentals i de la Salut, Universitat Pompeu Fabra, Doctor Aiguader 88, Barcelona, Spain.

^2^ Genomics Core Facility, Departament de Ciències Experimentals i de la Salut, Universitat Pompeu Fabra, Parc de Recerca Biomèdica de Barcelona, 08003 Barcelona, Spain.

^3^ Department of Immunology, Hospital Clínic, Barcelona, Spain.

^4^ Institut d’Investigacions Biomèdiques August Pi i Sunyer (IDIBAPS), Barcelona, Spain.

^5^ Catalan Institution of Research and Advanced Studies (ICREA), Passeig de Lluís Companys, 23, 08010, Barcelona, Spain.

^6^ CNAG-CRG, Centre for Genomic Regulation (CRG), Barcelona Institute of Science and Technology (BIST), Baldiri i Reixac 4, 08028 Barcelona, Spain.

^7^ Institut Català de Paleontologia Miquel Crusafont, Universitat Autònoma de Barcelona, Edifici ICTA-ICP, c/ Columnes s/n, 08193 Cerdanyola del Vallès, Barcelona, Spain.

^8^ Universitat de Barcelona, Barcelona, Spain

**Supplementary material**

**Supplementary figures**


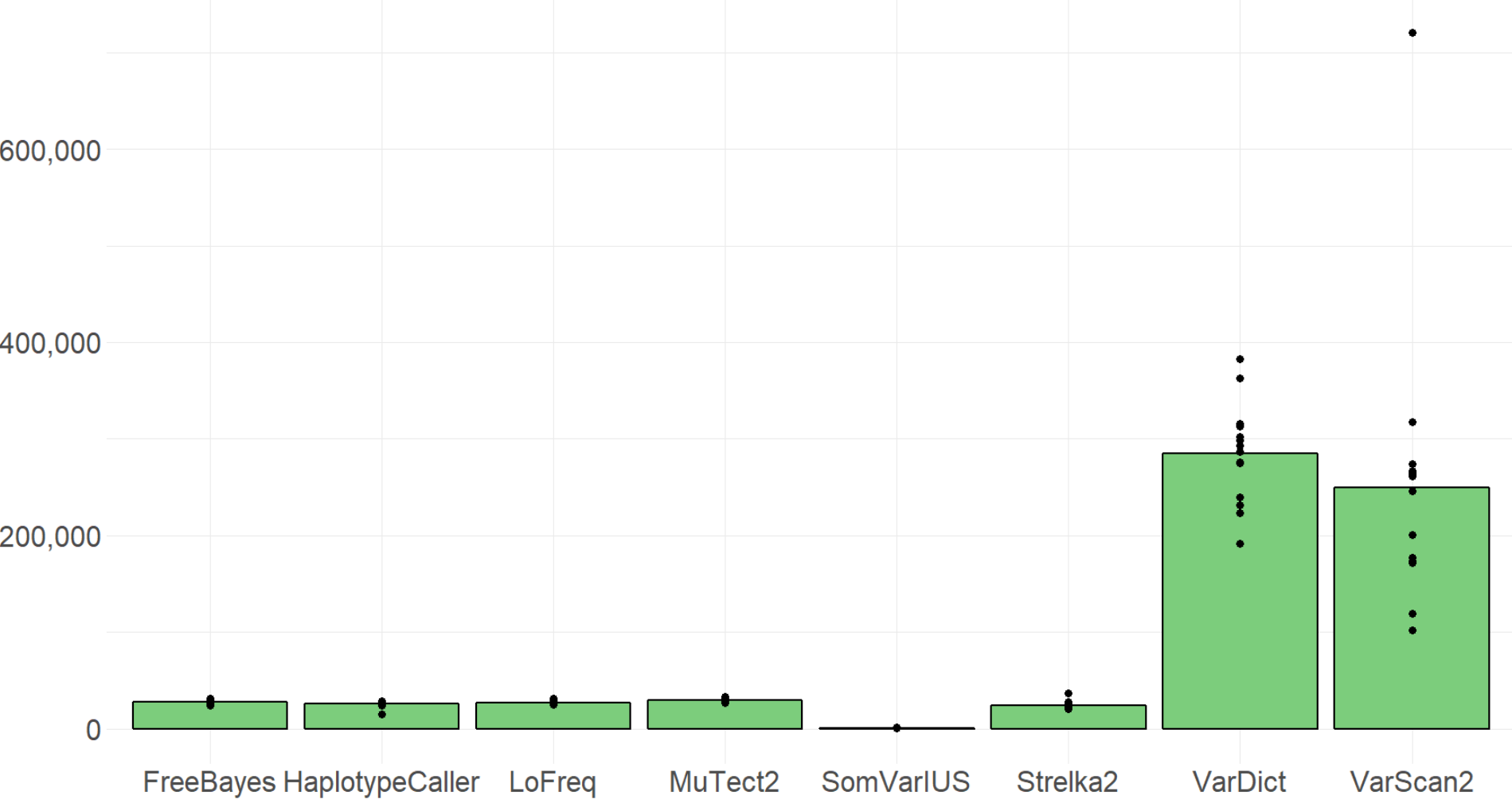


**Supplementary Figure 1.** Mean number of mutations detected per variant caller in on target regions. Each dot represents each one of the whole blood samples.


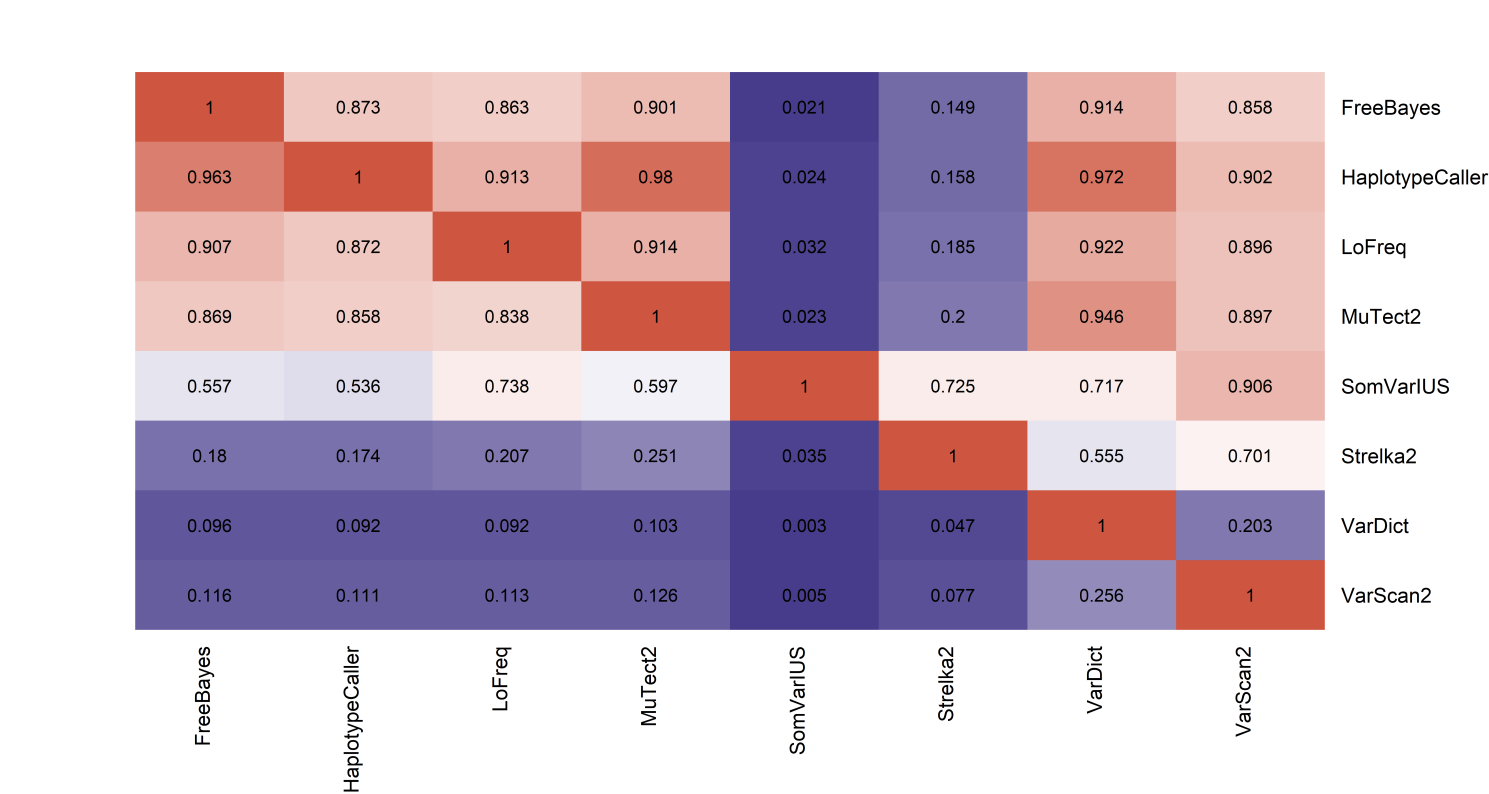


**Supplementary Figure 2.** Percentage of overlapping calls among variant callers in blood samples.


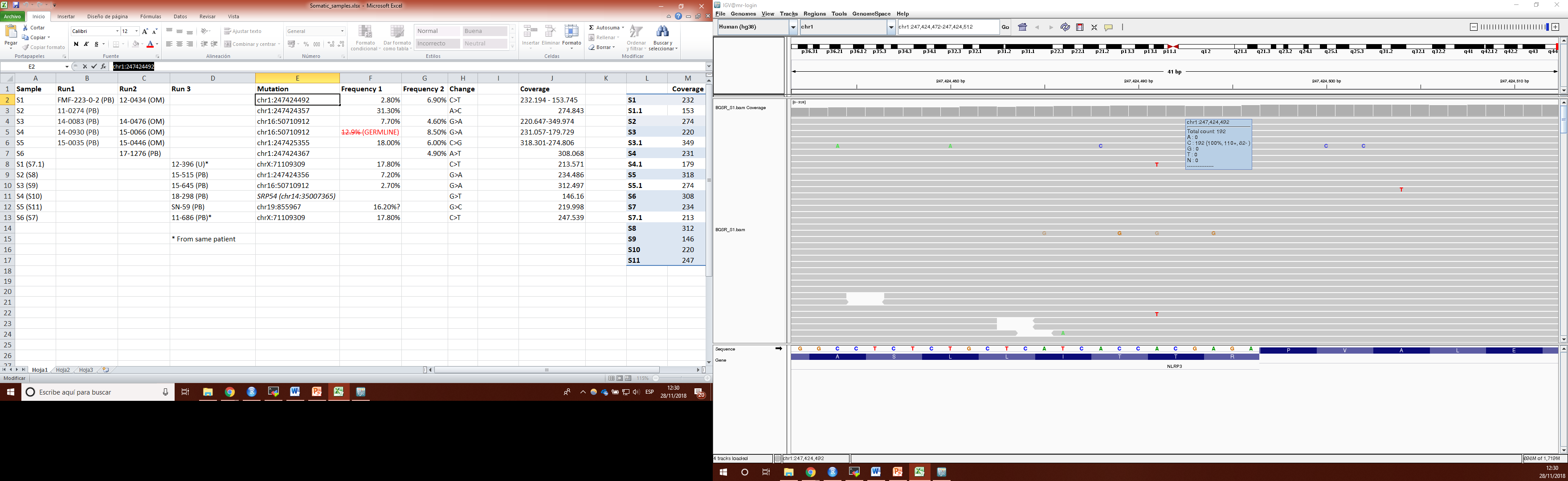


**Supplementary Figure 3.** IGV screenshot of the region including the somatic mutation in S1a. We observe no reads supporting the alternate allele.


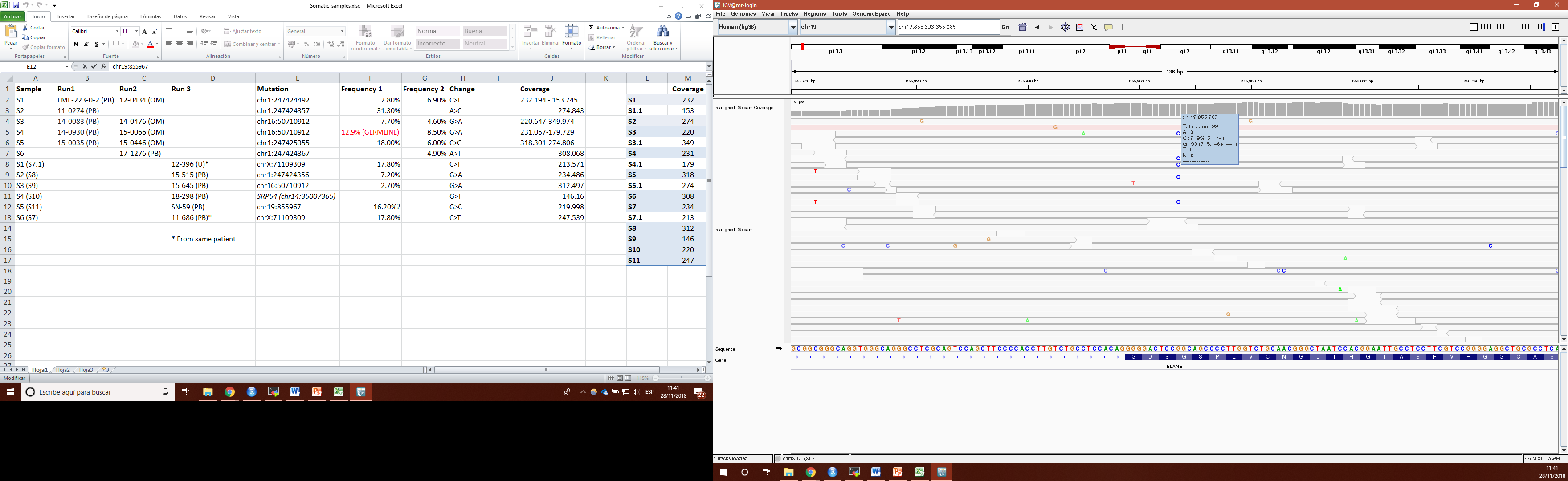


**Supplementary Figure 4.** IGV screenshot of the region including the somatic mutation in S11. It is only called by VarDict and VarScan2.


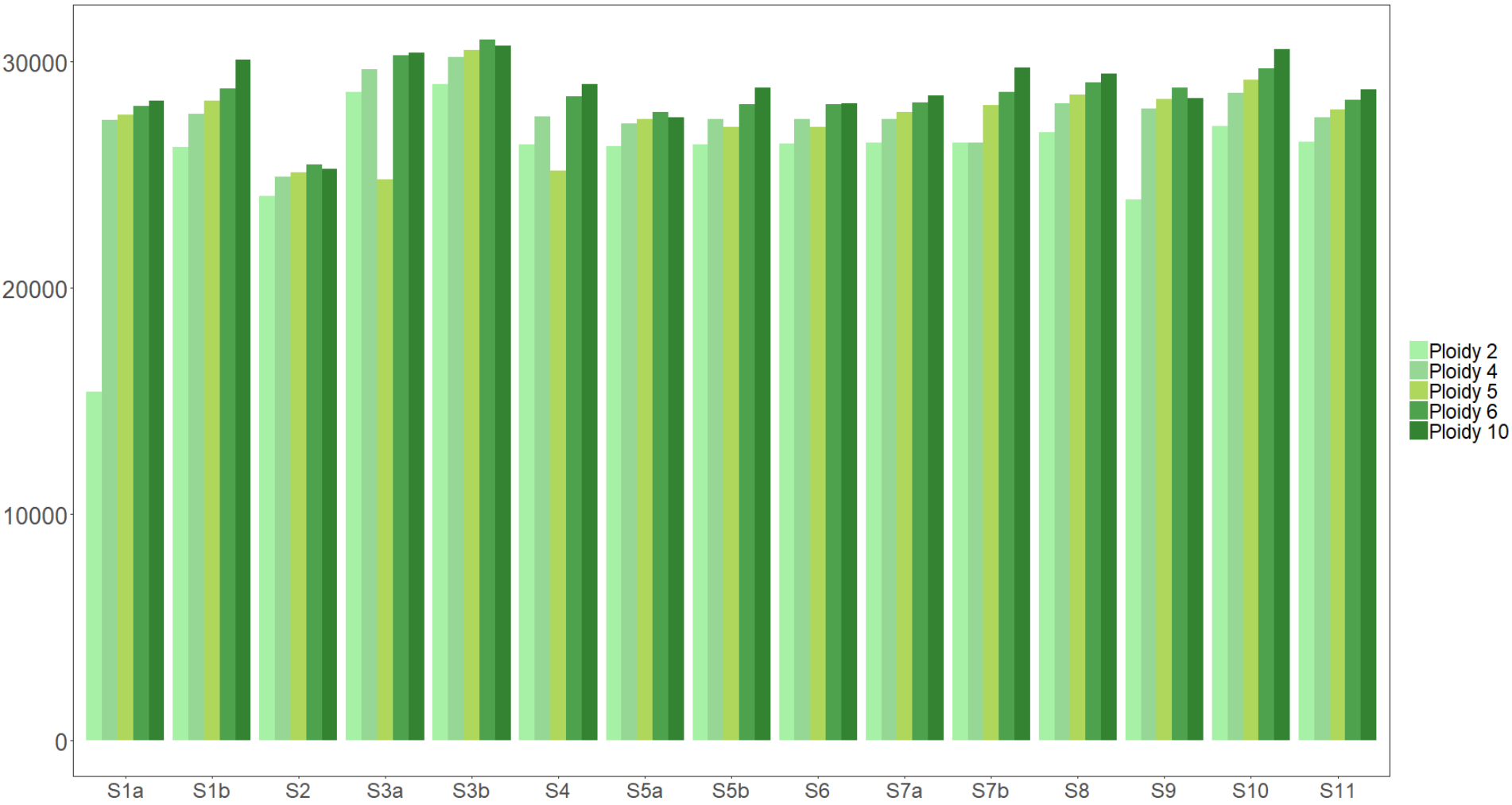


**Supplementary Figure 5.** Number of on target calls for HaplotypeCaller with ploidy 2, 4, 5, 6 and 10 in blood samples.

We can observe that, in some cases, the number of variants called decreases when increasing the ploidy. This is due to the fact that our high coverage introduces more sequencing error, which is contemplated in the calling and, due to memory reasons; GATK does not compute some qualities in the gVCF files with default parameters. So, when obtaining the genotypeGVCFs, those positions are ignored. This behaviour can be avoided by setting --max_num_PL_values parameter to a higher one. Interestingly, this is the case for one of our variants, which is not called with ploidy 10 (S5b). Beware of this default behaviour of HaplotypeCaller.


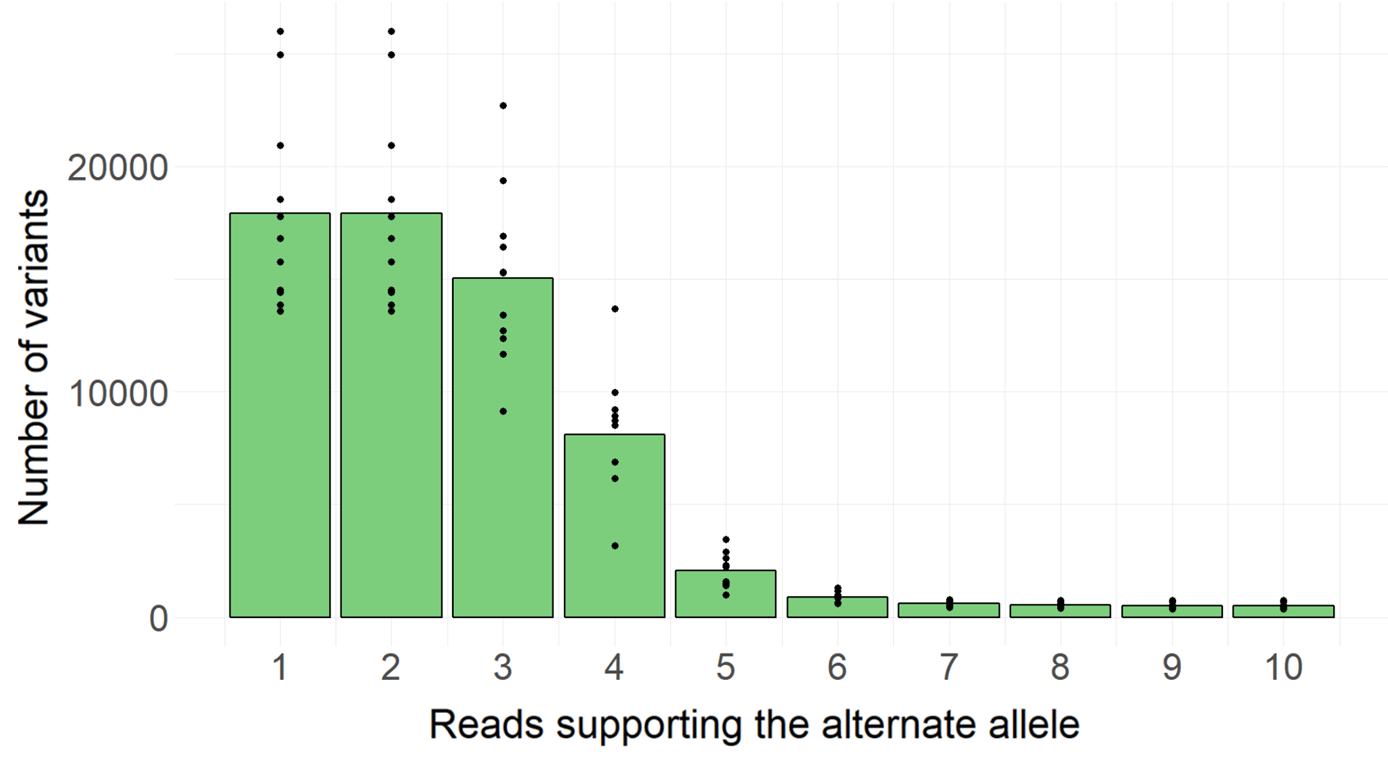


**Supplementary Figure 6.** Mean number of variants per individuals with, at least, the numbers in the x-axis of reads supporting the alternate allele. We see a clear decline and this number is stabilized at 7. Each dot represents each one of the whole blood samples.

**Supplementary tables**

|  | LoFreq | MuTect2 | Strelka2 | VarDict | VarScan2 |
| --- | --- | --- | --- | --- | --- |
| S1a-S1b | NO/NO | NO/NO | NO/YES | YES/YES | YES/YES |
| S3a-S3b | NO/NO | YES/NO | NO/NO | YES/YES | YES/NO |
| S5a-S5b | NO/NO | NO/NO | NO/NO | YES/YES | YES/YES |
| S7a-S7b | NO/YES | YES/NO | YES/NO | YES/YES | YES/YES |

**Supplementary Table 1.** Detected (YES) and non-detected (NO) mutations in paired mode for the 5 callers with this mode available. We used first in the comparison the first sample (S1a, S3a, S5a and S7a) as tumour and the second (S1b, S3b, S5b and S7b) as normal, and then in the inverted order.
